# Assessment of Family Tuberculosis Contact Screening Practice and its Associated Factors Among Pulmonary Tuberculosis Positive Patients in South Wollo Zone, Amhara Region, Ethiopia

**DOI:** 10.3389/ijph.2023.1605815

**Published:** 2023-06-15

**Authors:** Tadesse Jember, Getachew Hailu, Gizachew Tadesse Wassie

**Affiliations:** Department of Epidemiology and Biostatistics, School of Public Health, College of Medicine and Health Science, Bahir Dar University, Bahir Dar, Ethiopia

**Keywords:** screening, Ethiopia, tuberculosis, family contact, pulmonary TB index cases

## Abstract

**Objectives:** The objective of this study was to assess the prevalence and the associated factors of family contact screening practice.

**Methods:** An institution-based cross-sectional study was conducted among 403 randomly selected pulmonary tuberculosis index cases from 1st May to 30th June 2020. Data were collected through a face-to-face interviewer-administered questionnaire. Multivariable logistic regression was performed.

**Results:** The prevalence of family contact screening was 55.3%, (CI: 60–50). Having family support for care and treatment (AOR = 2.21, 95% CI: 1.16–4.21), waiting time of less than 60 min (AOR = 2.03, 95% CI: 1.28–3.21), receiving health education on TB prevention and treatment (AOR = 1.86), 95% CI: 1.05–3.29), and having good knowledge about TB prevention (AOR = 2.76, 95% CI: 1.77–4.294) were factors associated with family TB contact screening practice.

**Conclusion:** This study revealed that the prevalence of family contact screening was low as compared to national and global targets. Factors associated with family contact screening practice were: the presence of family support, shorter waiting time, health education offered by healthcare workers, and a good level of knowledge of the index cases.

## Introduction

Contact screening is a key component of a tuberculosis (TB) control program [1] because TB is transmitted from person to person [2]. The initially identified case of new or recurrent TB in a person of any age in a specific household or other comparable settings in which others may have been exposed is an index case [3]. TB contact is “anyone who has had recent contact with someone who has active TB” [4].

Contact cases are categorized for their risk of getting the disease and their relationship with the pulmonary TB index cases and/or those having developed active TB are classified as household contact (HC), non-household close contact (NHCC), causal contacts, and community contacts [5]. HCs are those who share the same household or other enclosed environments as a person who is known or suspected to have TB and are classified as the highest risk group to develop TB [6]. NHCCs are people who have had regular, extensive contact with the index case and share breathing space daily or almost daily but do not sleep in the same household most of the time. NHCCs can include caregivers, regular sexual partners, close friends, extended family, daycare and primary/secondary school classroom contacts, and co-workers that work in close physical proximity (particularly in small rooms). Regular contacts in specialized healthcare settings such as dialysis units or rehabilitation programs may also qualify [7, 8].

TB is a disease that affects the lungs (pulmonary tuberculosis (PTB)), but it can affect other body sites other than the lungs (Extra pulmonary tuberculosis), and the causative agent of TB is *Mycobacterium tuberculosis* [9, 10]. It is a communicable disease and a major cause of morbidity and mortality as it is among the top ten causes of death from a single infectious agent globally [11].

Latent tuberculosis (LT) is also an infection with Mycobacterium (bacterium) without any signs of clinically or radiologically manifest disease and is usually referred to as a suspected infection with a bacterium tuberculosis complex [10]. The diagnosis of LT is based on a tuberculin skin test reaction [10]. Active case searching through family contact screening with early case identification in the community and giving effective treatment using a Directly Observed Treatment Short-course (DOTS) improves the TB infection control program. HCs are highly vulnerable to acquiring TB from the index cases because of their proximity [12].

Each year, over 3 million TB cases are undiagnosed globally due to the gap in case identification. This leads to a persistent risk of infection from the index case for the family and communities as a whole without any investigation. One way of identifying the index cases is through screening the families or HCs for TB [13].

Contact tracing starts as soon as a TB case is identified. Those who might have been in contact with infected individuals are asked to be screened for TB to reduce transmission [14]. Sputum smear-positive pulmonary TB patients are given priority in household and close contact investigations for TB in the national guidelines of Ethiopia and other countries. HC screening is influenced by social, cultural, spiritual, and other socio-demographic factors and these factors are essential for the contact investigation program to be successful [3, 15].

In Ethiopia, the prevalence of adhering to family contact screening was indicated to be very low when compared to the stated targets globally as well as nationally [16]. The feat of HC screening not only results in the tracing of cases but it has several advantages in early diagnosis, reducing the rate of morbidity, reducing the risk of transmission to others, and enhancing high yield; hence HCs are the priority for TB screening [12, 17, 18].

Although Ethiopia is among the 30 high TB burdened countries and among the 14 triple (TB, TB/HIV, MDR-TB) burdened countries [19], and all the HCs of all PTB index cases should be screened, the prevalence of missed undiagnosed PTB cases in the community is very high [20]. Besides, among pulmonary index cases, the prevalence of and determinants for family contact screening are not yet well known. Therefore, this study aimed to assess the prevalence and the associated factors of family contact screening practice in the study area.

## Methods

### Study Design and Period

An institutional-based cross-sectional study design with primary data from a structured interviewer-based questionnaire was conducted for PTB-positive patients. Data were collected at the DOTS centers with PTB-positive cases in public health facilities in the South Wollo Zone from 1st May to 30 July 2020.

#### Study Area and Setting

South Wollo is one of the administrative zones of the Amhara regional state zone with twenty-one woredas, one City administrative zone, three town administrative woredas, and one special ethnic administrative district with 588 kebeles. It has an estimated total population of 3,346,166. Of this, 1,534,842 are males [21]. In South Wollo there are 713 public health facilities (one referral hospital, 11 primary hospitals, 129 health centers, and 572 health posts) and 35 private health facilities (five general hospitals, 30 medium and primary hospitals). Directly Observed Therapy service (DOTs) is delivered by 129 public health centers and 10 hospitals in the South Wollo Zone, and by two public hospitals and seven health Centers in the Dessie city administration. There were a total of 508 estimated PTB cases in the zone reported in the half report from July 2011 to December 2012 EFY.

#### Study Population

The source population was pulmonary TB patients who were registered in DOTs clinics from all public health facilities in the South Wollo Zone and who were on treatment during the study period with any phase of treatment from 1st May to 30 June 2020.

#### Sample Size Determination

The sample size was calculated with a single population proportion formula to estimate the total sample size using a proportion from a previous study done in North Gondar Town health facilities, in Northwest Ethiopia, which was 47.5% [22], and the assumption that the index cases brought their families for TB screening with a 95% confidence interval and 5% margin of error.

$$n=\frac{{Z\alpha ⁄2}^{2}.p.\left(1-p\right)}{d^{2}}$$

$$n=\frac{1.96^{2}\left(0.457\right)\left(1-0.475\right)}{\left(0.05\right)2}$$

$$n=\frac{1.96^{2}\left(0.475\right)\left(0.525\right)}{\left(0.05\right)2}$$

n=383.19≈384

Considering a 10% no-response rate, the total sample size was ≈422.

#### Sampling Procedure

There are 21 administrative districts and three city administrations with 148 public health facilities providing DOTs in the South Wollo Zone including the Dessie city administration. Primarily health facilities with PTB patients on treatment were selected to allocate the sample proportionally. Total cases in the zone were identified in the District Health Information Software (DHIS2) reports from the 2nd, 3rd, and 4th quarters up to 20 April 2020 from the respective health facilities. Then simple random sampling was applied to select the sample of 422 PTB cases from a total of 528 PTB cases from all health facilities.

#### Variables of the Study

##### Dependent Variable

Family contact screening.

##### Independent Variables

Socio-Demographic characteristics: Age, sex, marital status, religion, occupation, educational status, family size, monthly income, family support, age of the family contacts, sex of the family contacts, and residency are included.

Health system-related characteristics: Variables included in the health system are the type of health facility, time taken to get to the HF/TB clinic, mode of transportation to the TB clinic, health education by healthcare workers, waiting time at TB clinic, service satisfaction, and cost of TB diagnosis.

Comorbidity of the index cases: HIV co-infection

Anti-TB treatment and care of the index case: disclosure status of the index case to families and anti-TB treatment outcome (improved, same, worsened).

##### Operational Definitions

Index case/patient: The first patient that has been diagnosed with new or recurrent TB in a specific household or other comparable settings in which others may have been exposed, irrespective of age [3].

Contact: Any person who has been exposed to an index case (as defined above) [3].

Household contact: A person who shared the same enclosed living space for one or more nights or for frequent or extended periods during the day with the index case 3 months before the commencement of the current treatment episode.

Close contact: A person who is not in the household but shared an enclosed space, such as a social gathering place, workplace, or facility, for extended periods during the day with the index case during the 3 months before the commencement of the current treatment episode.

Family contact screening: PTB index cases were considered as having practiced contact screening if they brought at least one family contact to the TB clinic and screened for TB(Yes) and otherwise considered as not screened (No) [16].

Contact investigation: A systematic process intended to identify previously undiagnosed cases of TB among the contacts of an index case.

Waiting time: The time that is taken to get service after arrival at the health facilities (waiting time is expected to be less than an hour).

Knowledge: PTB index cases were classified as having good knowledge if they answered at least 80% of the 10 knowledge about TB questions but poor if they answered less than 80% of those questions [16, 22].

#### Data Collection Tool

Data were collected from PTB patients at the DOTs clinics of public health facilities in the South Wollo Zone by using a structured face-to-face interviewer-based questionnaire prepared in English and translated to an Amharic version. The family matrix of the contact screening logbook information was collected before the data collection and cross-checking was done before the data entry into Epi Info 7.

#### Data Quality Assurance

The questionnaire was developed in the English language and was translated to the Amharic version, which ensured data quality. The Amharic version was also translated into the English version. The data collection was run by 21 district health office officers other than the TB program runners who participated in the data collection and six supervisors were selected with the eligibility criteria of having the ability to write and read, language understanding level, and their previous exposure to data collection. Training was given for supervisors and data collectors about the data collection procedures. The questionnaire was pre-tested using approximately 5% of the sample in Woldia town health centers and Woldia Hospital, which gave the same service for TB, before starting the actual data collection. The collected data were also continuously reviewed for accuracy and completeness by supervisors and principal investigators accordingly.

#### Data Processing and Analysis

The data were entered into Epi Info version 7 and exported to SPSS statistical software version 23. The data were then coded, entered, cleaned, and analyzed using SPSS. Results were presented by percentage, frequency tables, and summary statistics. The socio-demographic characteristics of the PTB index cases such as marital status, age, sex, religion, educational status, family size and family support, comorbidity, treatment, and care-associated factors information were determined by binary logistic regression analysis. Factors of the level of family contact screening were explored by multivariable analysis. All variables in the bivariable analysis with a *p*-value less than 0.2 were entered into multivariable models using enter logistic regression. The model goodness of fit was checked using the Hosmer-Lemeshow test (HL test) and the result was *p*-value = 0.123. The variables held in the models reached a significance level of *p*-value <0.05. The degree of association between the independent and dependent variables was assessed using crude and adjusted odds ratios, and their statistical significance was assessed at a 95% confidence interval.

## Results

### Socio-Demographic Characteristics

A total of 403 PTB cases on treatment were interviewed from 422 expected cases with a 95% of response rate. Of this 241 (60.4%) were males and the mean age of participants was 35.41 (standard deviation of +12.97). In total, 233 (57%) of the participants were from rural residences (Table 1).

**TABLE 1:** Socio-demographic characteristics of respondents, S/Wollo, Ethiopia July 2020, (*n* = 403).

Variables	Categories	Frequency	Percentage (%)
Age	<20	48	11.9
	20–29	96	23.8
	30–39	127	31.5
	40–49	69	17.1
	>50	63	15.6
Sex	Male	243	60.3
	Female	160	39.7
Residency	Urban	170	42.2
	Rural	233	57.8
Marital status	Single	109	27.0
	Married	266	66.0
	Divorced	16	4.0
	Widowed	12	3.0
Education	No formal education	160	39.7
	Primary school	120	29.8
	Secondary school	71	17.6
	College and above	52	12.9
Occupation	Non-governmental employee	24	6.0
	Private	44	10.9
	Student	56	13.9
	Housewives	75	18.6
	Daily laborer	43	10.7
	Merchant	53	13.2
	Farmer	85	21.1
	Governmental employee	23	5.7
Position in the HH	Member	198	49.1
	Head	205	50.9
Monthly income in USD	<5.5	72	17.9
	5.51–11.00	105	26.1
	11.10–16.50	94	23.3
	>16.51	132	32.8
Family support	Yes	355	88.1
	No	48	11.9

### Health System and Patient-Related Factors

Approximately 94.5% of the participants were treated at health centers. More than half (65.8%) of the participants got to a TB clinic after more than half an hour’s journey on foot and 30.5% of them also had access to public transport. Approximately 96% of the respondents were satisfied with the health service given in the TB clinic. Almost 61.8% of the respondents reported that they stayed in the health facility for more than an hour. More than half (59.3%) of the participants scored sufficient knowledge and 93% of them received health education by the healthcare workers (Table 2).

**TABLE 2:** Health system and patient-related characteristics on pulmonary positive TB cases in South Wollo Amhara region, Ethiopia, 2020 (*n* = 403).

Variables	Categories	Frequency	Percentage (%)
Type of health facility	Hospital	15	3.7
	Health center	381	94.5
	Health post	7	1.7
Distance	>30 min	265	65.8
	<30 min	138	34.2
Mode of transportation	On foot	208	51.6
	With animals	72	17.9
	With public transport^a^	123	30.5
Health Education by HCW	Yes	327	81.1
	No	76	18.9
Waiting time	>60 min	249	61.8
	<60 min	154	38.2
Service satisfaction	Yes	386	95.8
	No	17	4.2
Knowledge on TB	Poor	164	40.7
	Good	239	59.3
HIV/AIDS comorbidity	Yes	26	6.5
	No	377	93.5
Phase of treatment	Intensive	78	19.4
	Continuation	325	80.6
TB Disclosure status to families	Yes	383	95.0
	No	20	5.0
Treatment outcome	Improved	350	86.8
	The same	21	5.2
	Worsen	4	1.0
	Unknown	28	6.9

^a^ Public transport like Bajaj and Taxi.

### Prevalence of Family Contact Screening

In total, 223 (55.3%) of the (95% CI: 50%, 60%) index cases practiced contact screening. A total of 1,370 family members were reported from the total of 403 index cases. Of those families, 640 (46.7%) family members were screened for TB, and 23 (3.59%) of the screened contacts were reportedly positive for TB.

#### Factors Associated With the Practice of Contact Screening

In the bi-variable logistic regression analysis, the factors associated with contact screening at a *p*-value of <0.2 were average monthly income, family support, waiting time, the distance of the house of the index cases from health facility on foot, health education, and the level of knowledge of the index cases. Socio-demographic factors such as age, sex, educational status, occupation, marital status, and residency were not significantly associated with family TB contact screening practices using a simple logistic regression model and were not entered into the final regression model. In the multivariable analysis, family support, waiting time, health education by HCWs, and the level of knowledge the index cases have about TB were significantly associated with family contact screening of pulmonary positive index cases with a *p*-value of <0.05 and 95% CI. Thus, the odds of the index cases with family support were two times more likely to screen their families for TB than those who did not (AOR = 2.21, 95% CI: 1.16, 4.21). Those who experienced a waiting time in the health facilities for service access of less than 60 min were two times more likely to screen their families for TB as compared to those who had to wait for more than 60 min (AOR = 2.03, 95% CI: 1.28, 3.21). The index cases who received health education from healthcare workers were two times more likely to screen their families than those who did not receive it (AOR = 1.86), 95% CI: 1.05, 3.29). Also, the index cases who had good knowledge about TB were three times more likely to screen their families than those who did not (AOR = 2.76, 95% CI: 1.77, 4.294) (Table 3).

**TABLE 3:** Factors on the level of family contact screening among pulmonary tuberculosis patients using multivariate logistic regression, Amhara region, Ethiopia, 2020, (*n* = 403).

Variables	Category	Screening status		COR (95% CI)	AOR (95% CI)
		Yes	No		
Income (SUD)	<5.5	33	39	1	1
	5.51–11.00	56	49	0.53 (0.29, 0.95)	0.57 (0.30, 1.07)
	11.10–16.50	53	41	0.72 (0.42, 1.21)	0.92 (0.52, 1.61)
	>16.51	81	51	0.81 (0.47, 1.39)	0.94 (0.52, 1.70)
Family support	No	41	17	1	1
	Yes	182	163	2.16 (1.18, 3.9)	2.21 (1.16, 4.21)
Distance	>30 min	61	77	1	1
	<30 min	162	103	1.98 (1.30, 3.01)	0.64 (0.40, 1.02)
Waiting time	>60 min	157	92	1	1
	<60 min	88	66	2.27 (1.51, 3.42)	2.03 (1.28, 3.21)
Health Education	No	52	24	1	1
	Yes	171	156	1.97 (1.16, 3.35)	1.86 (1.05, 3.29)
Knowledge	Poor	114	50	1	1
	Good	109	130	2.71 (1.78, 4.13)	2.76 (1.77, 4.29)

## Discussions

Since contacts with PTB cases are categorized as high risk in the progression of TB disease [4, 23], in the early detection of TB disease, contact tracing is the primary activity, especially for close contacts and household/family contacts. Thus, the objectives of this study were to determine the prevalence of family contact screening and identify factors influencing the level of family contact screening. This study revealed the level of family contact screening was 55.3%. This was almost similar to the study in the rural area of hospital Shashemane, Ethiopia in 2013 (55.7%) [24]. However, this finding was higher than the findings reported in the studies in North West Ethiopia, in 2015, Gondar town in 2019, and in Tigray region in 2020, which were 33.7%, 47.5%, and 21.7%, respectively [16, 22, 25]. The possible reasons for this variation could be the low socio-economic status of the index cases and the lack of follow-up for contact screening in low-income countries. Adherence to the screening of household members is affected by individual, social-cultural, accessibility, and health system factors.

A possible reason for the low family contact screening rate may be due to low awareness about TB screening, and the advantages and disadvantages of screening for TB. Some study reports previously [16, 22, 26–28] indicate that screening adherence in family contact was influenced by income, awareness of TB, housing, and health system factors.

In this study, health system and patient-associated factors including family support were the most important factors; the index cases with family support were two times more likely to screen their families for TB. But this was inconsistent with the study in north Weast Ethiopia, which was the opposite as those with medium and no support were more likely to screen their families [16].

Health education given by healthcare workers about TB was also an important factor for the screening of the families of the index cases, as patients who received health education were two times more likely to initiate family contact screening. In our setting, health education focuses on signs and symptoms of TB, the advantage of early screening, TB infection prevention techniques, and the type of person they should bring to healthcare facilities for screening.

Similarly in Northwest Amhara, Ethiopia, receiving health education was positively associated with household contact screening for TB [16]. This was also similar to the study conducted in Gondar town [22]. Both studies showed that health education that focuses on the TB mode of transmission, prevention, and treatment may enhance family contact screening adherence rate and health-seeking behavior. Participants who had good knowledge about TB prevention and control strategies were three times more likely to screen their families than those who had poor knowledge. Previous articles have reported that poor knowledge is a barrier to service delivery in TB diagnosis, treatment, and prevention [29, 30].

The participants’ waiting time was significantly associated with family contact screening practice. Participants who waited at the health facility for less than 60 min were two times more likely to screen their families than those who waited for more than 60 min. Overcrowding, professional unpunctuality, a lack of commitment to service delivery, and sometimes repeated appointments brings increased waiting times at health facility [28]. In this situation, clients might be dissatisfied and unwilling to bring their family contacts to screen for TB.

### Limitations of the Study

A few study participants were non-responsive, although we considered a 10% = non-respondent rate while we calculated the sample size. And also due to the cross-sectional nature of the study, it did not allow for the determination of causation.

### Conclusion

The prevalence of family contact screening in the study area was low as compared to the national and the World Health Organization (WHO) targets in which all index cases were expected to screen their families. Factors associated with family contact screening in this study were family support, waiting time, health education offered by healthcare workers, and the level of knowledge of the index cases. Hence, advocating for index cases to disclose their status to their family or caregivers, enhancing service provision quality by minimizing waiting time, and practicing health education about TB prevention and control strategies in the primary healthcare service would be important strategies to increase family contact screening utilization. This research shows that there are gaps and factors that lead to the low TB screening practice which may hinder TB elimination efforts. In turn, further research should be conducted to evaluate why the current routine community surveillance system regarding family contact screening practices in the national TB control program failed to achieve the national target. Finally, conducting research to identify possible area-specific clusters of unscreened TB contacts may be much more beneficial.

## Data Availability

The datasets used and/or analyzed during the current study are available from the corresponding author on reasonable request.

## Abbreviations

AOR, adjusted odds ratio; AIDs, acquired immunodeficiency syndrome; COR, crude odds ratio; DOTs, directly observed therapy; HC, household contact; HIV, human immunodeficiency virus; LT, latent tuberculosis; MBT, *Mycobacterium tuberculosis*; MDR-TB, multi drug-resistant tuberculosis; NHCC, non-household close contact; PTB, pulmonary tuberculosis; SDG, sustainable development goal; TB, tuberculosis; WHO, world health organization; ZHD, zonal health department.
